# Masked Feature Residual Coding for Neural Video Compression

**DOI:** 10.3390/s25144460

**Published:** 2025-07-17

**Authors:** Chajin Shin, Yonghwan Kim, KwangPyo Choi, Sangyoun Lee

**Affiliations:** 1 School of Electrical and Electronic Engineering, Yonsei University, Seoul 03722, Republic of Korea; chajin@yonsei.ac.kr (C.S.); ywkimblue@yonsei.ac.kr (Y.K.); 2 Samsung Seoul R&D Campus, Seoul 06765, Republic of Korea; kp5.choi@samsung.com

**Keywords:** neural video compression, deep learning, residual, mask, feature, conditional coding

## Abstract

In neural video compression, an approximation of the target frame is predicted, and a mask is subsequently applied to it. Then, the masked predicted frame is subtracted from the target frame and fed into the encoder along with the conditional information. However, this structure has two limitations. First, in the pixel domain, even if the mask is perfectly predicted, the residuals cannot be significantly reduced. Second, reconstructed features with abundant temporal context information cannot be used as references for compressing the next frame. To address these problems, we propose Conditional Masked Feature Residual (CMFR) Coding. We extract features from the target frame and the predicted features using neural networks. Then, we predict the mask and subtract the masked predicted features from the target features. Thereafter, the difference is fed into the encoder with the conditional information. Moreover, to more effectively remove conditional information from the target frame, we introduce a Scaled Feature Fusion (SFF) module. In addition, we introduce a Motion Refiner to enhance the quality of the decoded optical flow. Experimental results show that our model achieves an 11.76% bit saving over the model without the proposed methods, averaged over all HEVC test sequences, demonstrating the effectiveness of the proposed methods.

## 1. Introduction

As higher-resolution videos are captured by camera sensors, the strain on device storage has increased significantly. Consequently, video compression has become important for reducing file size while preserving visual quality. To reduce this load, various standardized video compression technologies have been developed, which significantly reduce file sizes while maintaining video quality. Traditional video compression standards, such as H.264 [1], H.265 [2], VP9 [3], AV1 [4], and VVC [5], include numerous hand-crafted methods like block partitioning, inter and intra prediction, transformation, in-loop filter, and entropy coding. However, since each module is optimized individually and cannot leverage complex nonlinear operations, these methods are considered sub-optimal.

**Figure 1:**
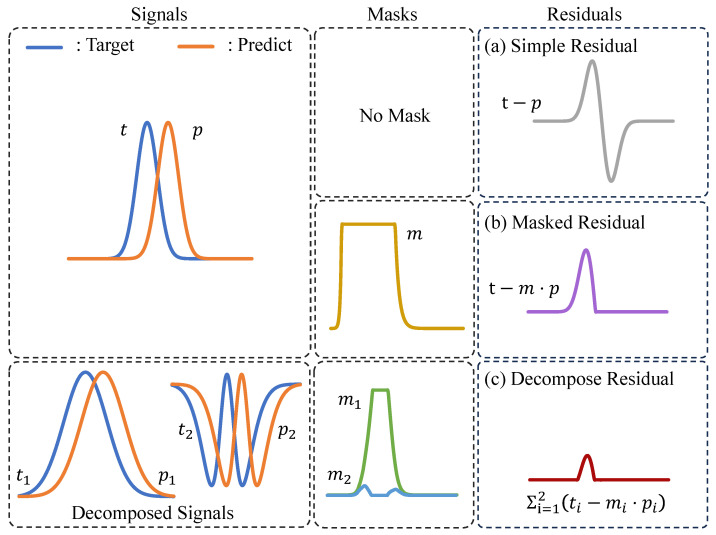
Upper Left: Target and Predict signals. Bottom Left: Decomposing each signal into low and high frequencies. To obtain the low-frequency signals, t1 and p1, we utilize a mean filter, while we subtract the low-frequency signals from the original signals to obtain the high-frequency signals, t2 and p2.

Recently, neural networks have shown significant performance improvements in many image and video tasks [6,7,8,9,10,11,12,13], and they have also been integrated into video compression. The notations and terminology related to neural video compression are listed in Table 1. DVC [14] replaces all modules in the traditional video codec with neural networks and trains the model in an end-to-end manner. DVC uses a simple subtraction operation to obtain the residual between the target frame ($x_{t}$) and the predicted frame ($x_{c}$). This can be demonstrated in $H(x_{t}-x_{c})$, which is referred to as Residual Coding. However, using a simple subtraction operation is not optimal. DCVC [15] uses $x_{c}$ as a conditional input along with the target frame into the encoder in the feature domain ($H(x_{t}|x_{c})$) which is called Conditional Coding. This allows the encoder to learn a more optimal operation to subtract the information in the predicted frame from the target frame. Brand et al. [16] assume that the entropy of $x_{t}-x_{c}$ is less than or equal to that of $x_{t}$, and show that Conditional Residual Coding ($H(x_{t}-x_{c}|x_{c})$) has less entropy than or equal to that of Conditional Coding. They feed the difference between $x_{t}$ and $x_{c}$ into the encoder along with the conditional input ($x_{c}$). However, a simple subtraction can result in a large residual. The upper left graph in Figure 1 shows an example of the target and the prediction signal. Then, we can obtain a (a) “Simple Residual” signal, which shows a large residual. Recently, Chen et al. [17] proposed Masked Conditional Residual Coding ($H(x_{t}-m⊙x_{c}|x_{c})$). They predicted a soft mask, applied it to the predicted frame ($x_{c}$), and then subtracted it from the target frame. This approach allows for a soft decision between using Conditional Coding and Conditional Residual Coding, which enables us to achieve smaller residuals. If we focus on the (b)“ Masked Residual” signal in Figure 1, assuming the mask is ideal, we can obtain a much smaller residual than that in (a). However, Masked Conditional Residual Coding has two limitations. First, applying a mask in the pixel domain and subtracting it from the target frame cannot consider the diverse and high-level representations in the images. Second, it is effective in reducing error propagation to use the reconstructed features as references to compress the subsequent frame. This is because abundant temporal context information [18,19,20,21] can be included in the reference features. However, Residual-type Conditional Coding methods require decoding the bitstream to the pixel level residuals (${\hat{x}}_{r}$) to obtain full information of the reconstructed target frame (${\hat{x}}_{t}={\hat{x}}_{r}+x_{c}$). Thus, they cannot utilize the reconstructed features of the previous frame as a reference.

To address these problems, we propose Conditional Masked Feature Residual (CMFR) Coding. As shown in the signals on the bottom left in Figure 1, the target and predicted signals can be decomposed into low and high frequencies. Then, by predicting different masks for each frequency component and applying them to the prediction signals, a much smaller residual can be obtained, as shown in (c) “Decompose Residual”. However, it is difficult to find the optimal components to extract for each content of the image, and the corresponding masks are also complex. Therefore, instead of using hand-crafted methods to extract the components from the image, we use a neural network to extract various features from the target frame and predicted features. We also predict the masks for each channel of the features using another neural network. Using these neural networks, our CMFR Coding can be expressed as $H(F_{t}\left(x_{t}\right)-m⊙F_{c}\left(x_{c}\right)|x_{c})$, where $F_{t}$ and $F_{c}$ represent the neural networks.

Moreover, we propose the Scaled Feature Fusion (SFF) module. This module predicts the fusion features and scales using conditional information along with the target frame information in the encoder or the residual information in the decoder. We apply the scales to the fusion features and subtract or add the scaled fusion features to the target or residual information, respectively. With this structure, conditional information can be effectively subtracted or added to the target or residual information within the context encoder and decoder. Furthermore, many methods of Conditional Coding [15,18,19,20,21] focus on accurately compensating the reference frame using the decompressed motion. However, they often overlook the quality of the decompressed motion. We propose a Motion Refiner module that uses reference features and the decompressed motion to improve the quality of the motion. Through various experiments, we demonstrate that the proposed methods significantly improve the rate-distortion performance of video compression.

Our main contributions can be summarized as follows:

- We point out the limitations of the existing Residual-type Conditional Coding, which masks and subtracts the prediction in the pixel domain.
- To resolve this problem, we propose a CMFR Coding, which extracts the features from the image followed by masking and subtraction in the feature domain.
- We propose the SFF module to effectively subtract or add conditional information to the target or residual information in the context encoder or decoder, respectively.
- We introduce the Motion Refiner module to enhance the quality of the decompressed motion vectors.

## 2. Related Works

Recently, deep learning techniques have demonstrated significant performance improvements in video processing, and there are many attempts to apply these techniques to neural video compression. Basically, they use a neural image compression structure [22,23,24] to compress the motion and the residual information. Lu et al. [14] propose the DVC model, which replaces all modules in the traditional codec with a neural network and trains the model in an end-to-end manner. They achieve a performance comparable to x265 [25] with the “veryfast” option, which is one of the fast encoding presets. They predict the motion between the reference frame (${\hat{x}}_{t-1}$) and the target frame, $x_{t}$, and compress it using the neural image compression structure [24]. Then, they predict the approximation of the target frame using the motion vector. Subsequently, the difference between the target frame $x_{t}$ and the prediction ($x_{c}$) is encoded using a simple subtraction operation in the pixel domain, referred to as Residual Coding ($H(x_{t}-x_{c})$). Using this residual structure, many papers [26,27,28,29] compress the residual between $x_{t}$ and $x_{c}$.

DCVC [15] highlights that a simple subtraction operation to obtain the residual is sub-optimal. To solve this problem, DCVC proposes Conditional Coding ($H(x_{t}|x_{c})$). DCVC extracts features from the reference frame and warps them in the feature domain to obtain the predicted feature of the current frame. Then, the target frame is fed into a context encoder, using the predicted feature as conditional input. With this approach, the context encoder can learn an optimized nonlinear function that subtracts the information in the predicted features from the target frame, achieving higher rate-distortion performance than the Residual Coding methods. Moreover, various works [18,19,20,21] that use Conditional Coding predict the approximation of the target frame in the feature domain at multi-scales, and utilize them as conditional input for the context encoder. This structure shows better performance than using only the predicted features of the original resolution.

However, the information bottleneck effect [30] can occur in the Conditional Coding approach due to information loss during the extraction of the features from the reference frame ($x_{t-1}$) to obtain the conditional input features of the context encoder. Consequently, it may not be possible to reduce the residual entropy to zero even if the result of motion compensation is identical to the target frame. To address this issue, Brand et al. [16] propose a new scheme to obtain the residual called Conditional Residual Coding ($H(x_{t}-x_{c}|x_{c})$). They assume that the entropy of $x_{t}-x_{c}$ is smaller than or equal to that of $x_{t}$, and demonstrate that Conditional Residual Coding ($H(x_{t}-x_{c}|x_{c})$) has less entropy than or equal to that of Conditional Coding. For processing the Conditional Residual Coding, they obtain the difference between $x_{t}$ and $x_{c}$ using a simple subtraction operation. Then, the difference is fed into the context encoder along with the conditional information, $x_{c}$. However, a simple subtraction operation can lead to large residuals, especially when the prediction of high-frequency components is incorrect.

To address this issue, Chen et al. [17] propose a new scheme called Masked Conditional Residual Coding ($H(x_{t}-m⊙x_{c}|x_{c})$). If the mask (*m*) has a value of 0, the encoder compresses the residual using Conditional Coding. In contrast, if the mask value is 1, the residual is encoded into the bitstream using Conditional Residual Coding. Therefore, they use a soft decision process with the mask to select which methods to use for encoding. With this structure, they achieve significantly better performance than other coding methods.

Meanwhile, Mentzer et al. [31] do not utilize motion information, and instead implicitly remove redundant information along the temporal axis using a transformer [32]-based entropy model. However, this process is performed only at the 1/16 scale. Lu et al. [33] use a hierarchical structure to implicitly reduce temporal redundancy. Nevertheless, the performance of their model is still lower than that of other state-of-the-art models because they also do not utilize motion vectors. Jia et al. [34] use implicit temporal modeling with simple convolutional layers and still outperform VVC by 21% in BD-rate. Jiang et al. [35] use both motion vectors and a transformer structure to capture correlations in the temporal context, particularly focusing on non-local correlations among the frames, and achieve a 38% bit saving on the UVG [36] dataset. Recently, bi-directional referencing has also been introduced in neural video compression models [37,38,39] to process the random access mode.

## 3. Method

In this section, we first describe the overall structure and then present the details of each proposed module.

### 3.1. Overall Structure

The goal of neural video compression is to compress the target frame by removing temporal redundancy with previous frames and encoding only the residual information. To do this, we estimate the motion vector ($v_{t}$) between the reference frame ($x_{t-1}$) and the target frame ($x_{t}$). We estimate $v_{t}$ using pre-trained SPyNet [40], which is widely used by existing neural video compression methods [14,15,18,19,20,21]. This motion vector is compressed and reconstructed (${\tilde{v}}_{t}$) using a motion encoder and decoder. This encoder has four convolutional layers with a kernel size of 3×3 and a stride of 2 for downsampling, and residual blocks consisting of two 3×3 convolutional layers with Leaky ReLU [41]. Moreover, the qp step (qs) is multiplied by the intermediate features at the last layer of each resolution for quality control. The qp step has the shape of $qs\in R^{1\times C\times 1\times 1}$, where *C* represents the channel size. The motion decoder has a symmetric structure with the encoder except that it uses convolutional layers with a kernel size of 1×1 followed by a pixel shuffle [42] for upsampling. Then, we employ a Motion Refiner module to enhance the motion vector. The refined motion vector (${\hat{v}}_{t}$) is used with the Multi-scale Context Fusion module to predict the approximation of the target frame in the feature domain at the original resolution, as well as at 1/2 and 1/4 resolutions ($C_{1\times }$, $C_{2\times }$, and $C_{4\times }$). We use a Temporal Context Mining (TCM) module from [18] as the Multi-scale Context Fusion module to predict ($C_{1\times }$, $C_{2\times }$, and $C_{4\times }$). The predicted features at the original resolution ($C_{1\times }$) are transformed and masked using a Mask Prediction module. Thereafter, the residual is obtained by subtracting these masked features from the features extracted from the target frame in the Residual Generator module. We compress this residual by feeding it into the context encoder, using the multi-scale predicted features as conditional input. In the context encoder and decoder, we introduce the SFF module to more effectively subtract information in the conditional input from the features of the target frame.

On the decoder side, we reconstruct the residual features (${\hat{r}}_{t}$) which are combined with $f_{sc}$ in the Combiner module to obtain the reconstructed features (${\hat{f}}_{t}$). Finally, we transform ${\hat{f}}_{t}$ into the reconstructed target frame (${\hat{x}}_{t}$) through a Pixel Mapping block composed of a single convolutional layer. ${\hat{f}}_{t}$ contains abundant temporal context information to prevent error propagation. Therefore, we store ${\hat{f}}_{t}$ and ${\hat{x}}_{t}$ in the reference buffer to utilize them when compressing the next frame.

We set the base channel size of the motion encoder and decoder to 64 for all resolutions. In contrast, the base channel sizes for all other modules are set to 48, 64, 96, 96, and 128 at 1/1, 1/2, 1/4, 1/8, and 1/16 resolutions, respectively. In addition, to obtain the qp step vector, we interpolate between the learnable parameters of $qs_{\max}\in R^{1\times C\times 1\times 1}$ and $qs_{\min}\in R^{1\times C\times 1\times 1}$ using the following equation:

(1) $$qs=\alpha \cdot qs_{\max}+\left(1-\alpha \right)\cdot qs_{\min}\;,$$

where α is the constant obtained by log-scale interpolation in the range from 0 to 1.

### 3.2. Motion Refiner

After estimating the motion vector between the reference frame and the target frame, we obtain the reconstructed motion vector (${\tilde{v}}_{t}$). Many Conditional Coding models [15,18,19,20,21] focus on warping and compensating the reference frame or features to accurately predict the target frame. They predict the features of $C_{1\times }$, $C_{2\times }$, and $C_{4\times }$, which are approximations of the target frame at the original resolution, 1/2 resolution, and 1/4 resolution, respectively. However, they often overlook the quality of the reconstructed motion vector.

The reconstructed motion vector (${\tilde{v}}_{t}$) often produces blurred edges around moving objects. We refine this motion before performing warping and compensation at the multi-scale level. First, we warp the reference feature using the reconstructed motion vector. Then, we use the motion vector and the warped features to compute $\Delta v_{t}$.

(2) $$\Delta v_{t}=V_{\theta v}\left({\tilde{v}}_{t},W\left({\hat{f}}_{t-1},{\tilde{v}}_{t}\right)\right),$$

where $V_{\theta v}$ denotes the neural network with learnable parameters θv, and *W* represents the backward warping operation. Subsequently, we obtain the refined motion vector (${\hat{v}}_{t}$) by adding $\Delta v_{t}$ to ${\tilde{v}}_{t}$.

(3) $${\hat{v}}_{t}={\tilde{v}}_{t}+\Delta v_{t}$$

Using this refined motion vector, we warp and compensate the reference features to predict the approximate features of the target frame ($C_{1\times }$, $C_{2\times }$, and $C_{4\times }$). We use these approximate features as conditional input for both the context encoder and the decoder.

### 3.3. Conditional Masked Feature Residual Coding

Masked Conditional Residual Coding [17] ($H(x_{t}-m⊙x_{c}|x_{c})$) is processed in the pixel domain. Therefore, it is not possible to apply different masks to the diverse and high-level representations of the image. To address this problem, we introduce CMFR Coding, which extracts features from the image, applies masking, and performs the subtraction.

First, we use a neural network to extract features from the target image ($F_{t}\left(x_{t}\right)$). Second, we predict the mask using a neural network. To predict the mask, we use the predicted features at the original resolution ($C_{1\times }$), the refined motion vector, and the warped reference frame.

(4) $$s=S_{\theta s}\left(C_{1\times },{\hat{v}}_{t},W\left({\hat{x}}_{t-1},{\hat{v}}_{t}\right)\right),$$

where $S_{\theta s}$ denotes the neural network with learnable parameters θs. Then, we map $C_{1\times }$ to $F_{c,C_{1\times }}$ using a mapping function ($F_{c}$), and use a tanh function [43] to mask $F_{c,C_{1\times }}$ as shown in the following equation:

(5) $$\begin{array}{cc} f_{sc} & =0.5\cdot [F_{c,C_{1\times }}+F_{c,C_{1\times }}⊙\tanh\left(s\right)]\\ \; & =0.5\cdot \left(1+\tanh\left(s\right)\right)⊙F_{c,C_{1\times }}\;, \end{array}$$

where ⊙ represents element-wise multiplication. On the context encoder side, the Residual Generator module is used to obtain the feature residual ($r_{t}$) by subtracting $f_{sc}$ from the feature extracted from $x_{t}$.

(6) $$r_{t}=F_{t}\left(x_{t}\right)-f_{sc}$$

Thereafter, we feed $r_{t}$ into the context encoder along with the conditional inputs predicted by the Multi-scale Context Fusion module.

(7) $$y_{t}=\mathrm{Enc}\left(r_{t},C_{1\times },C_{2\times },C_{4\times }\right),$$

where $y_{t}$ represents the latent vector. We refer to this residual encoding method as CMFR Coding.

(8) $$H(F_{t}\left(x_{t}\right)-m⊙F_{c}\left(x_{c}\right)|x_{c})$$

In contrast, on the Context Decoder side, we use the Combiner module to obtain the reconstructed features of the target frame. The Context Decoder predicts the reconstructed feature residual (${\hat{r}}_{t}$). Then, we add $f_{sc}$ to ${\hat{r}}_{t}$ and apply a residual block to obtain the reconstructed features.

(9) $${\hat{f}}_{t}=\mathrm{RB}1\left({\hat{r}}_{t}+f_{sc}\right),$$

where RB1 denotes the residual block consisting of two convolutional layers with a kernel size of 1×1 and a Leaky ReLU [41] activation function. Subsequently, we utilize a convolutional layer to transform ${\hat{f}}_{t}$ into the pixel domain, producing ${\hat{x}}_{t}$. This approach allows us to store the reconstructed features (${\hat{f}}_{t}$), which contain abundant temporal context information, in the reference buffer.

### 3.4. Scaled Feature Fusion

The context encoder transforms only residual information, which is the difference between the target frame and the conditional input, into the latent vector ($y_{t}$). To achieve this, we downsample the input features ($r_{t}$), and concatenate them with the predicted condition features ($C_{1\times }$, $C_{2\times }$, and $C_{4\times }$) at each resolution. To more effectively subtract conditional information from the input features, we introduce the SFF module.

**Figure 2:**
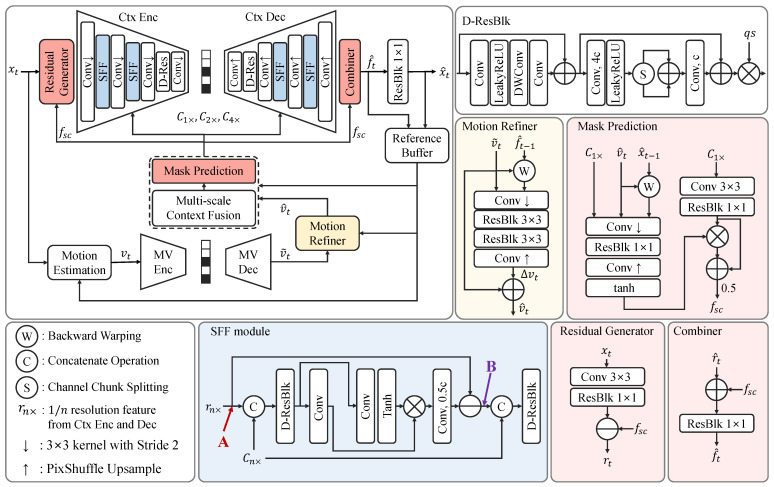
Overall structure of the network. The module diagrams colored in red, blue, and yellow represent the structures of our proposed Conditional Masked Feature Residual (CMFR) Coding, Scaled Feature Fusion (SFF) module, and Motion Refiner, respectively. “Conv” without any additional mark refers to a 1×1 convolutional layer, while “Conv, nc” represents the 1×1 convolutional layer with output channel size multiplied by a factor of *n*. “DWConv” denotes the depth-wise convolutional layer with a kernel size of 3×3. “ResBlk k×k” denotes the residual block consisting of two k×k convolutional layers with a LeakyReLU [41].

First, we predict the fusion features (*u*) and scales (*a*) using the input features and the predicted condition features as follows:

(10) $$\begin{array}{cc} u & =U_{\theta u}\left(r_{n\times },C_{n\times }\right)\\ a & =\tanh(A_{\theta a}\left(r_{n\times }\;,C_{n\times }\right)), \end{array}$$

where $U_{\theta u}$ and $A_{\theta a}$ represent neural networks to predict the fusion features and the scales, respectively. $r_{n\times }$ denotes the first features at 1/n resolution from the context encoder or decoder. These scales (*a*) operate through an attention mechanism, allowing it to focus on the regions that need to be subtracted effectively. Subsequently, we apply the scales to the fusion features and feed them into a convolutional layer to match the channel size of $r_{n\times }$. Then, we subtract the output of the convolutional layer from $r_{n\times }$, concatenate the result with $C_{n\times }$, and feed the combined features into a residual block.

(11) $$\begin{array}{cc} o_{1} & =r_{n\times }-\mathrm{Conv}\left(u⊙a\right)\\ o_{2} & =\mathrm{DRB}(\mathrm{Cat}\left(o_{1},C_{n\times }\right))), \end{array}$$

where Cat denotes the concatenation operation. DRB represents the depth-wise residual block, as depicted in Figure 2 (D-ResBlk). This module is used in both the context encoder and decoder at 1/2 and 1/4 resolutions.

## 4. Experiments

### 4.1. Implementation Details

To train our model, we use the Vimeo-90K [44] dataset, which consists of approximately 90,000 sequences, each containing seven frames. Each frame has a resolution of 448×256. During the training stages, we randomly crop frames to a size of 256×256 and apply random vertical and horizontal flips. Additionally, we randomly reverse the order of the frames in each sequence. We train our model with the multi-stage training strategy used in DCVC-TCM [18]. Unlike DCVC-TCM, we modify the training process in terms of compression quality control. We find that training our model becomes unstable when variable rate control modules are included from the beginning. Therefore, we first train our model at the highest video quality. After training at the highest quality, we incorporate variable rate control modules to enable training across varying compression levels. Then, to mitigate error propagation, we use a cascaded loss [18] to train the entire model on consecutive multi-frames.

In the training stage with multi-frames, we use six P-frames instead of the five that DCVC-TCM used. For the loss function, we use the Lagrange multiplier λ, which balances the quality of the reconstructed frame and the bit rate in a random range of [320,2600]. In addition, to reduce error propagation and maintain compression performance over long temporal sequences, we fine-tune our model using the BVI-DVC [45] dataset. This dataset consists of approximately 800 sequences at various resolutions. We fine-tune our model using 36 consecutive frames with cascaded loss [18] for 4 epochs at a learning rate of $1\times 10^{-5}$. Then, we decrease the learning rate to $1\times 10^{-6}$ for 1 epoch. For the MS-SSIM distortion, we use the $1-\text{MS-SSIM}({\hat{x}}_{t},x_{t})$ distortion function to train our model with λ values from 10 to 81.

### 4.2. Evaluation

To evaluate the video compression performance of each model, we use the UVG [36] dataset, which consists of seven sequences at a resolution of 1920×1080. Moreover, we use HEVC Class A to E [46], which have resolutions of 2560×1600, 1920×1080, 832×480, 416×240, and 1280×720, respectively. Additionally, we utilize the MCL-JCV [47] dataset. This dataset consists of 30 sequences with a resolution of 1920×1080. For all test datasets, we apply reflection padding to each input sequence to ensure that the width and height of the frames are multiples of 64. We use an intra period of 32 and a total of 96 frames for compression during the test.

To compare the rate-distortion performance of our model, we use HEVC [2], VP9 [3], and VVC [5] for traditional codec methods. In the case of HEVC, we use the HEVC reference software (HM) [48] version 18.0. For VP9, we use FFmpeg [49] version 7.0 with libvpx-vp9 of version 1.14.0. In the case of VVC, we use the VVC test model (VTM) [50] of version 23.4. For neural network-based P-frame video compression methods, we compare our model with DCVC [15], DCVC-TCM [18], DCVC-HEM [19], and DCVC-DC [20]. To evaluate rate-distortion performance, we measure PSNR and bits per pixel (BPP) and plot these values on a graph. Therefore, the graph on the left is a better model, as it uses fewer bits for the same visual quality. Furthermore, we utilize the BD-rate [51] to confirm the average bit savings achieved at the same visual quality level.

### 4.3. Rate-Distortion Performance

**Figure 3:**
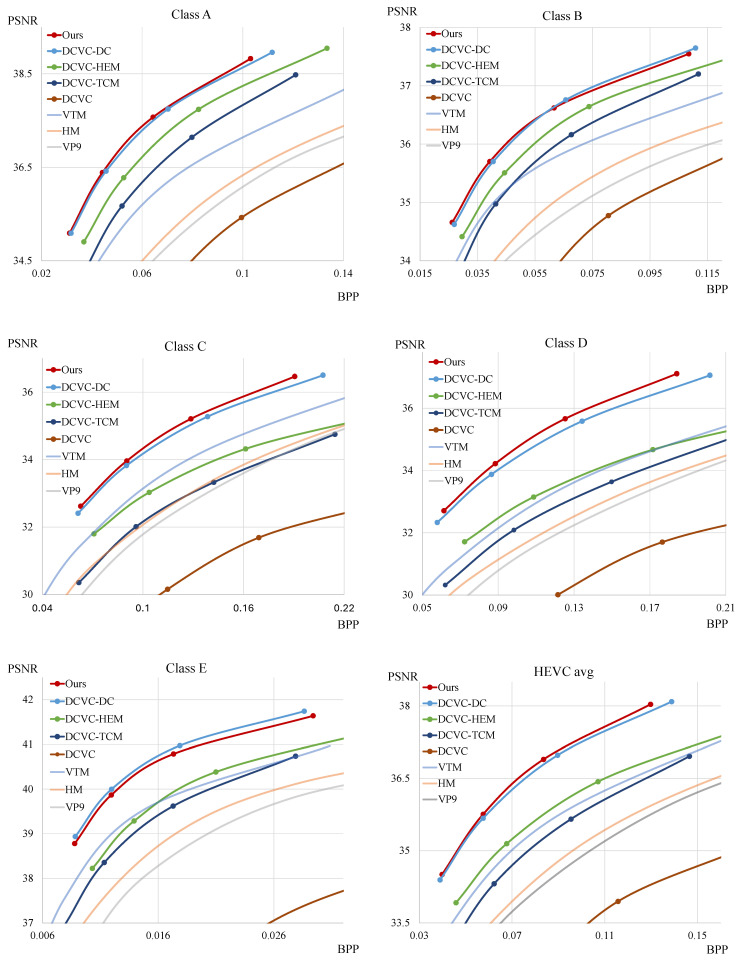
Rate-distortion performance comparison for different test sequences against several models.

**Figure 4:**
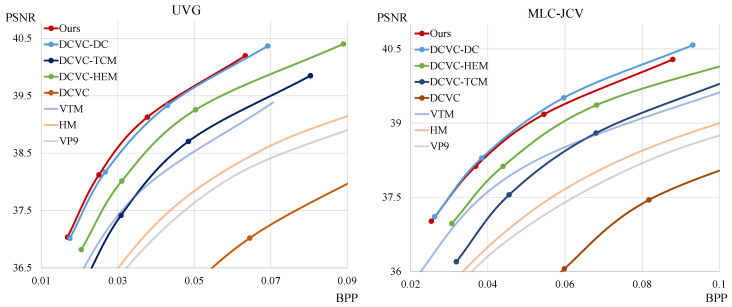
Rate-distortion performance comparison for different test sequences against several models.

Figure 3 and Figure 4 show the rate-distortion curves. The x-axis and y-axis represent the bpp and PSNR, respectively. Our model outperforms all traditional video codec methods, DCVC, DCVC-TCM, and DCVC-HEM, across all test sequences. Moreover, our model demonstrates higher compression performance in most sequences compared to DCVC-DC. Table 2 shows the BD-rate using VTM as an anchor with the PSNR distortion function, the total number of parameters, and the encoding and decoding time. To measure inference time, we use sequences with a resolution of 1920×1080 and average the results to obtain milliseconds per frame. For GPU measurements, we use the RTX A6000 Ada, while we utilize Ryzen 5 7500F for the CPU. The results indicate that our model demonstrates better compression performance than other models across most of the test sequences. Furthermore, compared to the DCVC-DC model, our model shows higher performance while requiring fewer parameters and less time for decoding. Table 3 represents the BD-rate with the MS-SSIM distortion function. As shown in the table, our model also achieves higher performance than others on most test sequences under the MS-SSIM metric.

**Figure 5:**
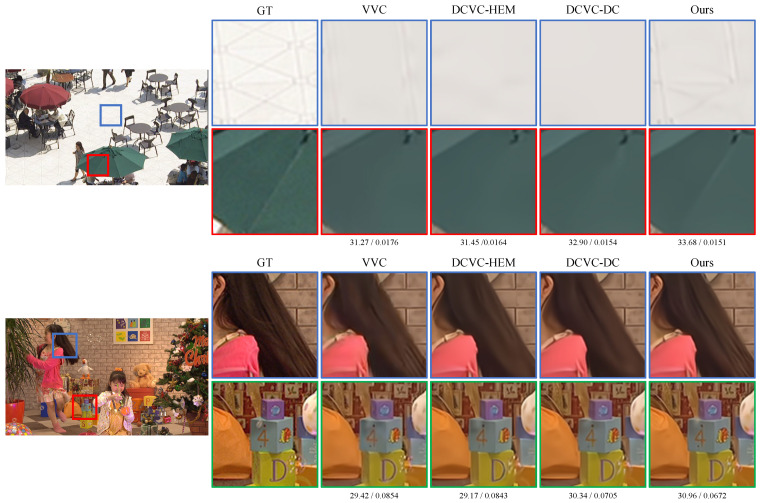
Qualitative comparison results with other SoTA neural video compression models and traditional codec. The values under the images indicate PSNR and BPP, respectively.

Figure 5 presents the qualitative comparison results. As shown in the upper image, our model preserves the pattern of the floor tiles better (blue box) and retains the folding lines of the parasol more clearly (red box). In the lower image, the texture of the girl’s hair is better preserved in our result (blue box). Moreover, the dot below the number 4 on the die is clearer than in the results of the others. In addition, the structure of the wrinkles on the orange bag is better maintained (green box).

## 5. Discussion

### 5.1. Conditional Masked Feature Residual Coding

**Figure 6:**
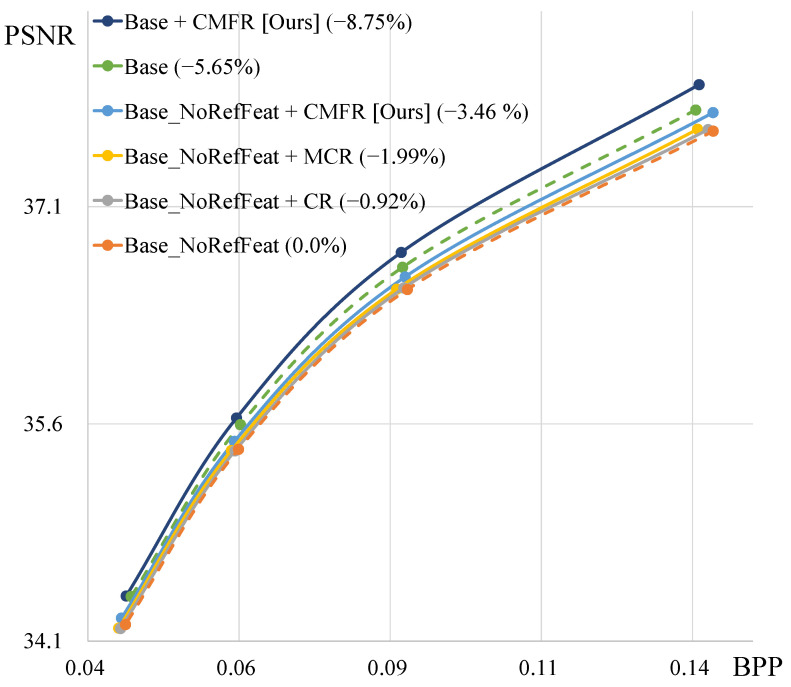
Ablation study results of various types of Conditional Coding on all HEVC test sequences. “NoRefFeat” indicates that we use the reference frame instead of features. “CMFR” denotes the proposed Conditional Masked Feature Residual Coding. “MCR” and “CR” represent Masked Conditional Residual Coding and Conditional Residual Coding, respectively.

**Figure 7:**
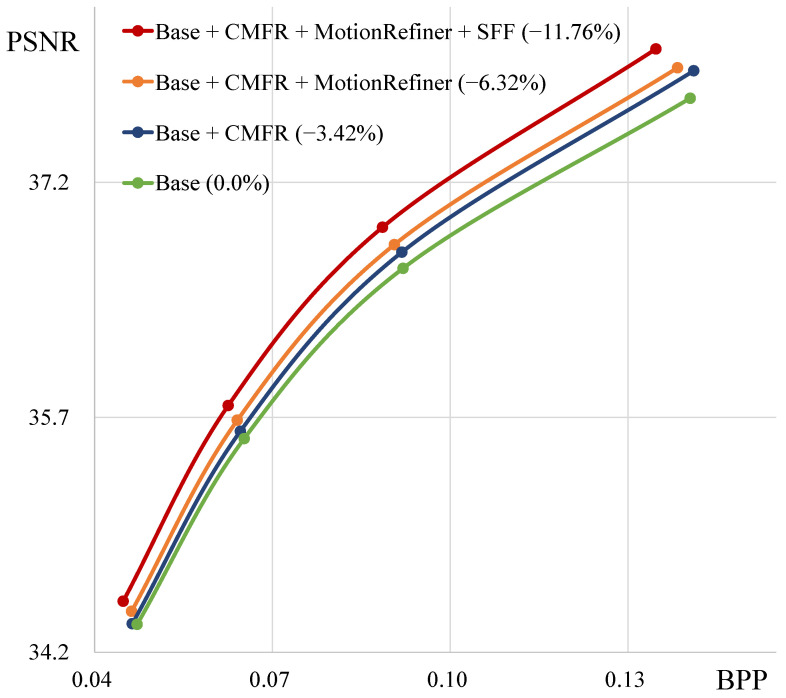
Ablation study result of each module on all HEVC test sequences. CMFR denotes the Conditional Masked Feature Residual Coding, and SFF represents the Scaled Feature Fusion module.

We compare our proposed method, CMFR Coding, with other Conditional Coding approaches. Figure 6 shows the rate-distortion curves of various coding methods. “Base_NoRefFeat” denotes the Conditional Coding model that uses reference frames instead of reference features. Therefore, this model fails to utilize abundant temporal context information. By applying Conditional Residual Coding or Masked Conditional Residual Coding to this base model, we find that the performance improves by 0.92% and 1.99%, respectively. When we apply our CMFR Coding to the base model, we observe a significant performance improvement of 3.46%. “Base” represents the base model, which uses reference features to leverage abundant temporal context information. Conditional Residual Coding and Masked Conditional Residual Coding cannot utilize this base model structure. In contrast, applying our method to both the “Base” and “Base_NoRefFeat” models demonstrates that CMFR Coding consistently improves rate-distortion performance. Table 4 and Figure 7 illustrate the performance improvements achieved by applying our proposed modules, along with the increases in model parameters and encoding/decoding times. Compared to base model A, model B from Table 4 achieves a performance improvement of 3.42% with a relatively small increase in the number of parameters. However, since CMFR Coding is applied at the original resolution, the inference time increases slightly.

**Figure 8:**
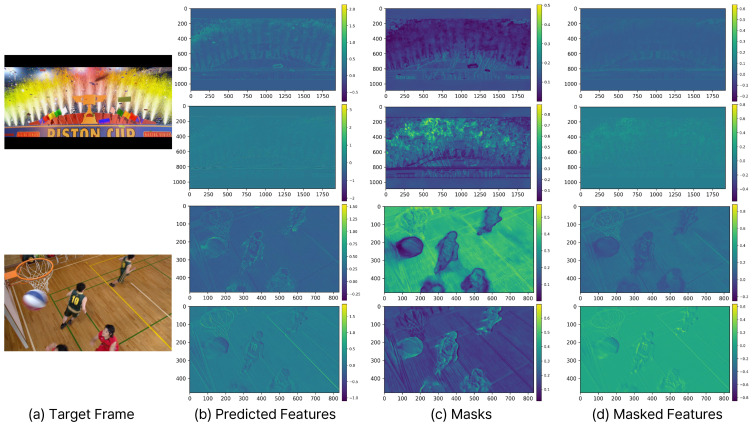
Visualization results of the masks and the masked predicted features from the Masked Prediction module from Figure 2 and Equation (5).

Figure 8 shows that the masks of our CMFR Coding are applied differently for each channel. (a) in Figure 8 shows the target frame, while (b) depicts the predicted features, which are $F_{c,C_{1\times }}$ in Equation (5). (c) illustrates the masks, defined as 0.5·(1+tanh(s)) in Equation (5). Finally, (d) presents the masked predicted features, denoted as $f_{sc}$ in Equation (5). These results indicate that the mask values change significantly at the boundaries of the moving objects. Moreover, although the spatial location is the same, the masks are applied differently across channels. For the same spatial location, some channels activate with high mask values, while others deactivate with low mask values.

### 5.2. Motion Refiner

**Figure 9:**
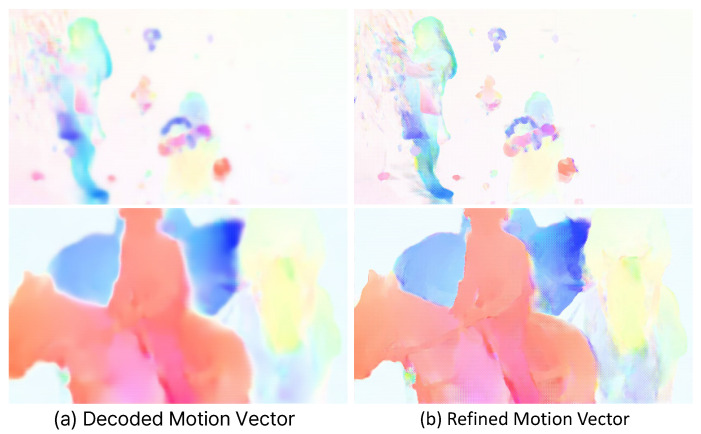
(**a**) Reconstructed motion vector, which is the output of the motion decoder. (**b**) Refined motion vector.

Figure 9 visualizes the results of the Motion Refiner module. This module refines the decoded motion vector using the reference features. (a) shows the decoded motion vector (${\tilde{v}}_{t}$) obtained through the decoder, and (b) depicts the refined motion vector (${\hat{v}}_{t}$). In (a), the edges of the moving object appear blurred. In contrast, the edges of the refined motion vector are clear and sharp. The rate-distortion plot in Figure 7 shows that the Motion Refiner module consistently improves video compression performance across the entire quality range. Furthermore, as shown in Table 4, this module achieves a performance improvement of 2.9%, while it requires only an additional 0.7 M parameters and increases decoding time by approximately 23 ms.

**Figure 10:**
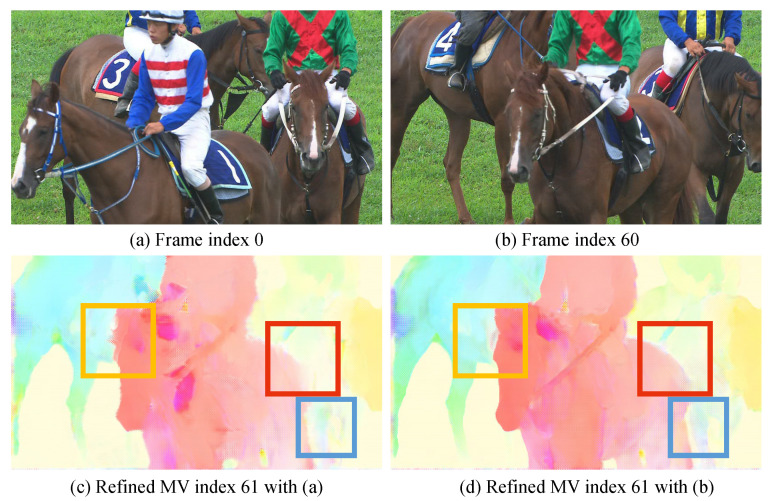
Motion refining analysis. (**a**) and (**b**) represent the frames with index 0 and 60, respectively. (**c**,**d**) depict the refined motion vectors for frame index 61. (**c**) uses the reference feature from (**a**), while (**d**) utilizes the reference feature from (**b**).

Figure 10 demonstrates how the neural network refines the motion vector using reference features. (a) and (b) represent the frames with index 0 and 60, respectively. Moreover, (c) and (d) depict the refined motion vectors for frame index 61. (c) uses the reference feature from (a), while (d) utilizes the reference feature from (b). Even if the structure of the target motion is not aligned with the reference frame, the edge shapes remain considerably similar. The neural network can use this characteristic to refine the edge or overall shape of the motion vector. As shown in Figure 10, the motion vector of frame index 61 has a similar edge shape with the previous frame (b). Therefore, (d) demonstrates a sharper edge and more accurate structure.

**Figure 11:**
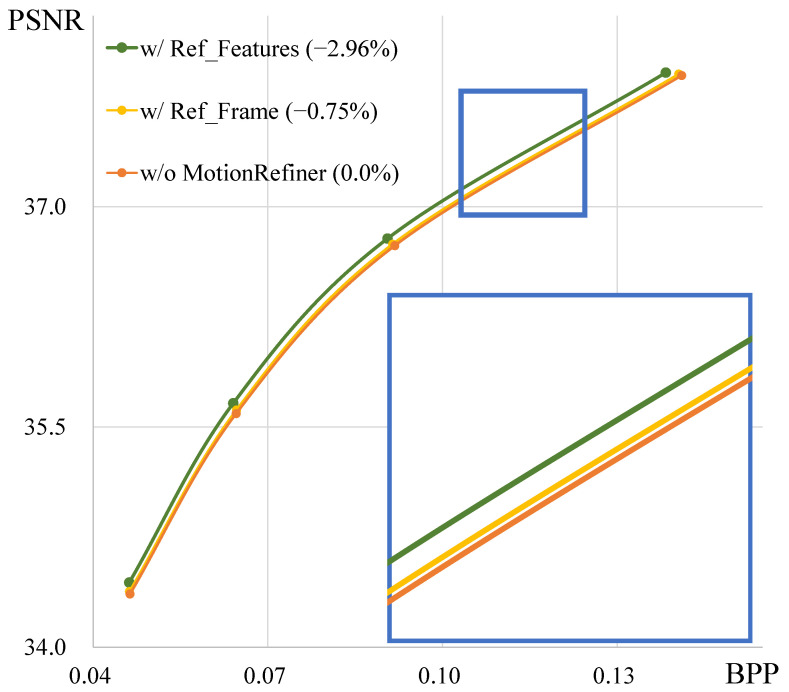
Rate-distortion results for motion refinement with reference features (w/Ref_Features) or reference frame (w/Ref_Frame) on all HEVC test sequences.

Figure 11 shows the results of predicting $\Delta v_{t}$ with reference features (${\hat{f}}_{t-1}$) or reference frames (${\hat{x}}_{t-1}$) in the Motion Refiner module. Using ${\hat{f}}_{t-1}$ to predict $\Delta v_{t}$ results in significantly better performance compared to ${\hat{x}}_{t-1}$. Therefore, we use reference features for motion refinement, as they are more effective.

### 5.3. Scaled Feature Fusion

As shown in Figure 7 and Table 4, the SFF module increases performance by 5.44%, with a parameter increase of about 1.14 M. Furthermore, Table 5 demonstrates that the SFF module effectively improves rate-distortion performance. This table shows the performance with the SFF module (“w/SFF”) and when it is replaced by three consecutive depth-wise residual blocks (“w/3 D-ResBlks”). These results indicate that the three consecutive residual blocks, which use more parameters than the SFF module, result in 1.46% performance increase. In contrast, our SFF module demonstrates 5.87% performance improvement while using fewer parameters.

**Figure 12:**
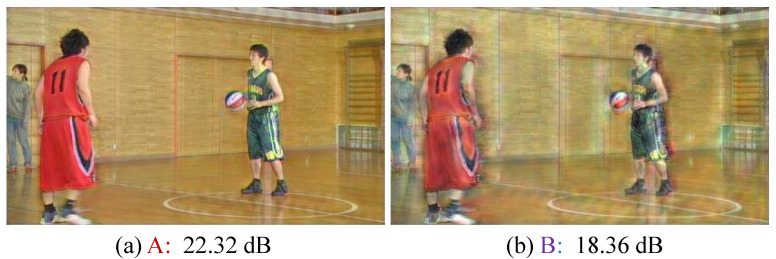
Visualization of features from the SFF module. We transform the features at point A (red) and B (purple) from the SFF module block diagram in Figure 2 into the pixel domain using additional decoder. (**a**) shows the feature at point A, and (**b**) shows the feature at point B, both transformed into the pixel domain.

We visualize the intermediate features from the SFF module. To do this, we select the first SFF module in the context encoder and define two points for visualization: point A (red) and point B (purple), as shown in the SFF module block diagram in Figure 2. Then, we freeze the pre-trained neural network and train only an additional decoder that transforms the features at points A and B into the pixel domain. This decoder consists of three and four residual blocks for points A and B, respectively, followed by a convolutional layer and a pixel shuffle layer. Using this additional decoder, we can visualize what information has been removed by transforming the intermediate features into the pixel domain. Through this process, we confirm whether the conditional information is actually subtracted from the target feature $r_{n\times }$ through the feature subtraction operation. Figure 12a shows a PSNR of 22.32 dB at point A of the SFF module in the pixel domain. After the subtraction operation with $r_{n\times }$, the PSNR is reduced to 18.36 dB at point B, as shown in (b). Moreover, as illustrated in Figure 12, the colors are distorted and many details are lost. These results confirm that the SFF module subtracts conditional information from the target feature $r_{n\times }$ through the subtraction operation.

### 5.4. Error Propagation

In this section, we evaluate the effect of error propagation and verify the effectiveness of using reconstructed features as references and fine-tuning the model with 36 consecutive frames. In the case of Residual Coding in the pixel domain, the decoder reconstructs the residual ($\hat{r}$) and then adds it to the predicted frame ($x_{c}$) to obtain the reconstructed frame (${\hat{x}}_{t}=\hat{r}+x_{c}$). However, ${\hat{x}}_{t}$, which has only three channels, loses abundant temporal context information. To cope with this problem, DCVC-TCM [18] stores reconstructed features in the buffer, which contain richer temporal information across multiple channels.

**Figure 13:**
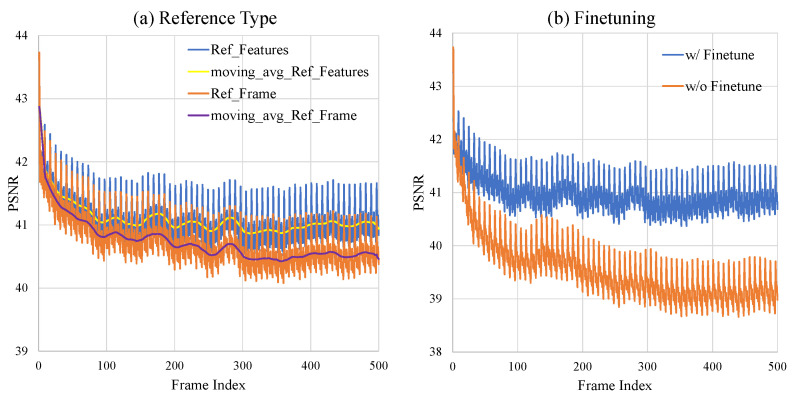
Visualization of error propagation effects. (**a**): Comparison between using reconstructed frames or features as reference. (**b**): Evaluating the effectiveness of fine-tuning on 36 consecutive frames using the BVI-DVC [45] dataset.

To verify this, we adapt the evaluation method for error propagation utilized in DCVC-FM [21]. We use “KristenAndSara” from the HEVC test sequence class E [46] as the test sequence. Only the first frame is set as an intra frame, and the remaining 499 frames are set as P frames to induce error propagation. We evaluate the “Base” and “Base_NoRefFeat” models from Figure 6, which use reconstructed features and frame as reference, respectively. Figure 13a represents the effect of the reference type on the intensity of error propagation. The x- and y-axes represent the frame index and the PSNR value, respectively. Moreover, “Ref_Features” and “Ref_Frame” correspond to the “Base” and “Base_NoRefFeat” models, respectively. The graphs labeled “moving_avg” indicate that a moving average with a window size of 16 is applied to the PSNR values along the frame index axis. As shown in Figure 13a, “Ref_Features” shows a decrease in PSNR until around frame 100, and then it remains stable in visual quality. In contrast, the PSNR of “Ref_Frame” continues to decrease, indicating more severe error propagation.

Moreover, the Vimeo-90K [44] dataset consists of seven frames per sequence, allowing a maximum of six consecutive P-frames to be used for training, as the first frame is used as the I-frame. This leads to a significant gap between training and test settings. Therefore, it is necessary to train the network under similar conditions to the test environment in order to effectively mitigate error propagation in long-term sequences. In Figure 13b, “w/o Finetune” refers to the model trained only on the Vimeo-90k dataset, while “w/Finetune” indicates the result of further fine-tuning this model using 36 consecutive frames from BVI-DVC [45]. As shown in this figure, “w/Finetune” shows stable PSNR from around frame 100, while “w/o Finetune” shows a continuous decrease in PSNR. This observation demonstrates that reducing the gap between the training and test environments effectively mitigates error propagation.

### 5.5. Limitations and Future Works

Although our proposed methods show performance improvements in terms of rate-distortion, several limitations remain. The first limitation is the memory size of the Decoded Picture Buffer (DPB). In the case of Conditional Residual and Masked Conditional Residual Coding, only 4×H×W×3 bytes are required for the size of the DPB when using 32-bit floating point. In contrast, feature-based method, including our CMFR Coding, store both the reconstructed frame and the feature maps. Therefore, when the channel size is 48, the required memory size is H×W×(3+48)×4 bytes, which is approximately 17 times larger than that of frame buffering methods. In terms of latency, there is no significant difference between storing the reconstructed frame and feature maps in terms of neural network structure. This is because both approaches transform the reconstructed frame or feature map into another feature domain using a convolutional layer and use it for motion compensation. In the case of data transfer over the bus, we measure the time required to transfer frame and feature maps from the CPU to GPU over 100 iterations. The total transfer time for frames is 0.1163 s, while the feature maps required 1.3621 s. This indicates that feature map transfer is approximately 11 times slower. However, if the reconstructed frame or feature is stored in the same device buffer as the neural network, the reference data fetching speed becomes much faster than the values we measured.

The second limitation is the large memory requirement in inference. We analyze how much GPU memory is required for decoding at commonly used resolutions. A resolution of 1280×720, which contains 921 K pixels, requires approximately 8.35 GB of video memory. For 1920×1080 (2073 K pixels), the memory usage increases to about 18.24 GB, and for 2560×1600 (4096 K pixels), it requires 34.81 GB. In the case of 4 K resolution, which is now widely adopted, approximately 70 GB of GPU memory is required, presenting a challenge for deploying neural video compression models to edge devices. To address this issue, one of the most straightforward approaches is to reduce the bit width of the data. Replacing 32-bit floating-point precision with 16-bit or 8-bit, along with fine-tuning [52], can significantly reduce both memory usage and inference time. Additionally, techniques such as pruning [53] could be utilized in future work to further reduce memory and computational costs.

The third limitation is the long decoding time. Table 2 presents a comparison of encoding and decoding time as well as KMACs between the proposed model, other SoTA neural video compression models, and traditional codecs. For traditional codecs, unlike the neural network models, KMACs vary depending on the content, so we measure only CPU-based inference time. As shown in the Table 2, our model achieves faster encoding speed than VVC when using either GPU or CPU. However, the decoding speed is approximately 10 times slower than VVC, even with GPU acceleration. Moreover, we observe that although DCVC-DC and our model show lower KMACs than DCVC-HEM and DCVC-TCM, their inference times are longer. This is due to the network architecture, which utilize numerous 1 × 1 convolutional layers and depth-wise convolutional layers, resulting in deeper networks. These operations have relatively low computational complexity individually. However, when they are stacked and proceed sequentially, the total inference time can significantly increase, as such a sequential process cannot benefit from parallelism or fast algorithms. As a result, the inference time becomes longer despite the lower KMACs.

This long inference time leads to a significant challenge for using neural video compression models in practical environments, despite their superior performance. Therefore, reducing decoding time is critical for future works. One of the main bottlenecks in decoding is arithmetic coding performed on the CPU and data transfer between the CPU and GPU. They account for approximately 20% (65.34 ms per frame) of the total decoding time. To address this, future work could focus on performing arithmetic coding directly on the GPU or designing new architectures that allow arithmetic coding and neural network computation to be processed in parallel.

## 6. Conclusions

In this study, we propose Conditional Masked Feature Residual (CMFR) Coding. With this approach, we can minimize the residual information between the target frame and the compensated features. For CMFR Coding, we utilize neural networks to extract the diverse and high-level representations from the target frame. Then, we predict masks and apply them to the predicted features to reduce the residual. Moreover, we introduce the Motion Refiner, which enhances the quality of the decoded optical flow. Additionally, we propose the Scaled Feature Fusion (SFF) module to more effectively remove and add conditional information in the context encoder and decoder, respectively. We demonstrate that our methods effectively minimize residual information and achieve a 36.28% performance improvement compared to VVC on the HEVC test sequences. Furthermore, we analyze limitations of the proposed model in terms of inference time, memory usage, and buffer size.

## Figures and Tables

**Table 1:** Summary of notations and terminology used in this paper.

Symbol	Full Name	Explanation
$x_{t}$	Target frame	The current frame to be encoded.
$x_{c}$	Compensated frame/Prediction	The frame predicted from previous frames with motion compensation.
*m*	Mask	A soft mask that determines how much of the predicted pixel or features to retain.
${\hat{x}}_{t}$	Reconstructed frame	The decoded current frame.
${\hat{F}}_{t}$	Reconstructed features	The decoded current features. This can be mapped to ${\hat{x}}_{t}$ with a neural network.
$C_{N\times }$	Conditional input	1/N resolution features predicted by the reference frame or features with motion vector.
${\hat{x}}_{t-1}$	Reference frame	The previously decoded frame used to encode the target frame.
${\hat{f}}_{t-1}$	Reference features	The previously decoded features used to encode the target frame.

**Table 2:** Rate-distortion performance comparison with other models across various test sequences on PSNR distortion. Inference time is measured using sequences with a resolution of 1920×1080.

Model	Dataset	VP9	HM	VTM	DCVC	DCVC-TCM	DCVC-HEM	DCVC-DC	Ours
BD-rate (%)	UVG	54.81	40.82	0.00	168.37	0.27	−19.74	−35.06	−37.90
	HEVC-A	48.77	36.67	0.00	85.81	−14.66	−27.74	−39.67	−41.09
	HEVC-B	65.06	44.72	0.00	131.66	−3.56	−18.49	−32.07	−33.13
	HEVC-C	40.99	30.76	0.00	162.16	33.04	11.53	−24.07	−27.48
	HEVC-D	39.08	29.83	0.00	136.30	12.97	−1.97	−37.01	−41.37
	HEVC-E	67.05	45.01	0.00	285.24	13.43	1.15	−33.68	−30.08
	HEVC-avg	46.69	35.65	0.00	146.61	10.21	−6.38	−34.28	−36.28
	MCL-JCV	57.74	42.94	0.00	139.63	10.40	−13.82	−29.35	−27.34
Parameters (M)		-	-	-	7.94	10.71	17.52	19.78	19.48
GPU Enc Time (ms/frame)		-	-	-	2438.34	374.15	318.33	464.75	482.93
GPU Dec Time (ms/frame)		-	-	-	12,345.73	230.37	192.01	359.74	334.15
CPU Enc Time (ms/frame)		307.89	8357.62	37,905.31	17,831.36	26,427.89	23,015.48	35,378.43	37,520.83
CPU Dec Time (ms/frame)		6.41	30.71	45.8	16,446.42	23,247.03	19,253.71	27,824.46	26,073.00
KMACs/pixel (Enc)		-	-	-	1180.38	1750.62	1700.87	1360.05	1490.21
KMACs/pixel (Dec)		-	-	-	776.26	933.10	1270.31	930.52	918.71

**Table 3:** Rate-distortion performance comparison with other models across various test sequences on MS-SSIM distortion.

Model	Dataset	VP9	HM	VTM	DCVC	DCVC-TCM	DCVC-HEM	DCVC-DC	Ours
BD-rate (%)	UVG	32.23	18.68	0.00	57.97	−12.22	−36.08	−40.44	−42.07
	HEVC-A	35.22	24.73	0.00	71.61	−0.15	−31.46	−38.18	−37.25
	HEVC-B	33.24	23.19	0.00	55.32	−15.12	−45.74	−51.15	−52.46
	HEVC-C	28.35	22.07	0.00	40.13	−17.88	−41.57	−53.48	−55.61
	HEVC-D	30.1	25.16	0.00	18.73	−31.02	−54.20	−61.91	−63.79
	HEVC-E	32.08	28.49	0.00	104.56	−28.67	−54.57	−59.83	−57.32
	HEVC-avg	32.84	23.41	0.00	62.48	−25.4	−41.29	−53.41	−56.41
	MCL-JCV	31.57	27.63	0.00	29.06	−11.77	−36.61	−48.75	−46.03

**Table 4:** Ablation study results of each proposed module on all HEVC test sequences. Inference time is measured using sequences with a resolution of 1920×1080 on the RTX A6000 ada.

	A	B	C	D
CMFR		✓	✓	✓
MotionRefiner			✓	✓
SFF				✓
BD-rate (%)	0.0	−3.42	−6.32	−11.76
Parameters (M)	17.41	17.64	18.34	19.48
GPU Enc Time (ms/frame)	332.79	398.66	411.18	482.93
GPU Dec Time (ms/frame)	212.48	275.73	298.03	334.15

**Table 5:** Ablation study result of Scaled Feature Fusion module on all HEVC test sequences. “w/SFF” represents the model with the SFF module, while “w/o SFF” denotes the model without it. In addition, “w/3 D-ResBlks” indicates the model where the SFF module is replaced with three consecutive depth-wise residual blocks.

Model	w/o SFF	w/SFF	w/3 D-ResBlks
BD-rate (%)	0	−5.87	−1.46
Parameters (M)	18.34	19.48	20.08

## Data Availability

Publicity available datasets were analyzed in this study [36,44,45,46,47].
